# Reduction of Thermal Residual Strain in a Metal-CFRP-Metal Hybrid Tube Using an Axial Preload Tool Monitored through Optical Fiber Sensors

**DOI:** 10.3390/polym14204368

**Published:** 2022-10-17

**Authors:** Zhao Li, Wei Ke, Mingyao Liu, Yang Zhou

**Affiliations:** 1 School of Mechanical and Electronic Engineering, Wuhan University of Technology, Wuhan 430070, China; 2 State Key Laboratory of New Textile Materials and Advanced Processing Technologies, Wuhan Textile University, Wuhan 430200, China

**Keywords:** metal-composite hybrid structure, residual strain, dynamic characteristic, optical fiber sensors, metal-CFRP-metal hybrid tube

## Abstract

Thermal residual strains/stresses cause several defects in hybrid structures and various studies have reported the reduction of residual strain. This paper describes a method for reducing thermal residual strains/stresses in metal-CFRP-metal hybrid tubes (MCMHT). The proposed axial preload tool provides two ways to reduce the thermal residual strains/stresses during the co-cure bonding process: pre-compressing of the metal layers and pre-stretching of the unidirectional carbon fiber reinforced polymer (CFRP) layers. An online measurement technique with embedded optical fiber Bragg grating (FBG) sensors is presented. Thermal residual strains are evaluated based on classical lamination theory with the assumption of plane stress. The theoretical calculations and measurement results agree well. Furthermore, the dynamic characteristics of the MCMHTs are tested. The results show that the reduction of residual strain increases the natural frequency of the MCMHT, but is detrimental to the damping capability of the MCMHT, which imply that the intrinsic properties of the metal-composite hybrid structure can be modified by the proposed axial preload tool.

## 1. Introduction

As a special hybrid structure, sandwich structure is formed by two thin, stiff, strong faces, such as metal or fiber-reinforced polymer (FRP) composites, with a lightweight core material, such as foam, honeycomb, FRP composites, balsa, etc. For meeting the high-quality needs of modern industry, it combines material science, functional design, intelligent sensing, and integrated manufacturing into an interdisciplinary concept [1,2]. The three-layered metal-FRP-metal sandwich structure has the potential for lightweight and high strength, vibration, and noise reduction in the automotive, rail transportation, marine, and aerospace industries. This metal-composite hybrid structure combines the superior durability of metals with the attractive properties of FRP composites, such as lightweight, high specific strength and stiffness, good damping capacity, and tailorable ability [3].

Many articles about metal-composite hybrid structures study theoretically and experimentally concerning connection performance between the metal parts and FRP composites [1,4], mechanical properties [5,6], impact resistance [7,8], durability [9,10], machinability [11,12], energy harvesting [13], etc. However, most of the research is conducted on plate and beam structures, and only a few papers refer to the circular tube with metal-FRP walls [14,15,16] and few papers are reported for square tube with metal-FRP-metal sandwich walls due to difficult fabrication. Meanwhile, there are a limited number of articles concerning the thermal residual strains/stresses in metal-composite hybrid structures. Therefore, this important issue still requires more understanding and knowledge, especially for complex structural components such as the metal-CFRP-metal hybrid tube.

The thermal residual strains/stresses in metal-composite hybrid structures are inevitably generated during the manufacturing process. The most important manufacturing process factors include differences in elastic properties and coefficients of the thermal expansion (CTE) of the FRP and metal layers, the cure cycle, and the tool-part interaction. The thermal residual strains/stresses cause several defects in hybrid structures such as transverse cracking and delamination, decrease the fatigue performance and dimensional accuracy, reduce the structure’s strength and modulus, and affect the natural frequencies and flexural stiffness [17]. Various studies have reported the reduction of residual strain, including changing the material composition of the hybrid structure [18], modifying the curing cycle [19,20,21,22,23], using special tools [24,25,26], post-stretching [27], microwave curing process [28,29], etc. The axial preload tool proposed in this paper for residual strain reduction targets hybrid structures based on unidirectional CFRP composite. The measurement methods can be divided into three categories: non-destructive, semi-destructive, and destructive [30]. As a non-destructive measurement method, the FBG sensor is used to monitor residual strain development during cure processing because it is small in diameter, precise, stable, easy integration, and anti-interference [31]. In this paper, the MCMHT with sandwich walls based on steel skins and unidirectional CFRP core is proposed and fabricated. The axial preload tool is proposed to reduce the thermal residual strains/stresses during the co-cure bonding process by pre-compressing of the metal layers or pre-stretching of the CFRP layers. The analytical model for evaluation of thermal residual strains is proposed based on classical lamination theory. The thermal residual strains are measured in real-time by the embedded FBG sensors. The modal testing results show the intrinsic properties of the metal-composite hybrid structure can be modified by the proposed axial preload tool.

## 2. Experimental Procedure

### 2.1. Material and Structure of the MCMHT

**Figure 1:**
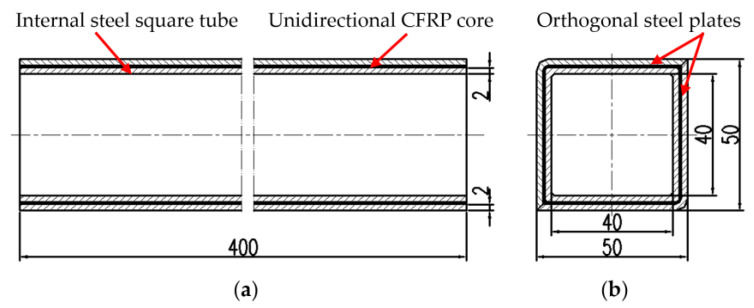
Structure and dimensions of the MCMHT: (**a**) main view; (**b**) left side view.

As shown in Figure 1, the MCMHT, with dimensions of 50 mm × 50 mm × 400 mm, is composed of an internal steel square tube, a square layer of unidirectional CFRP core, and two orthogonal steel plates. The unidirectional CFRP core in this paper consists of 10 layers of USN 10000/T300 prepreg from the Weihai Guangwei composites company with dimensions of 460 mm × 1000 mm × 0.1 mm. The material of the steel square tube is AISI 1045 based on the American Iron and Steel Institute (AISI) grade system. Material properties of the unidirectional CFRP prepreg and the steel square tube are listed in Table 1.

### 2.2. Fabrication Method of the MCMHT with the Axial Preload Tool

**Figure 2:**
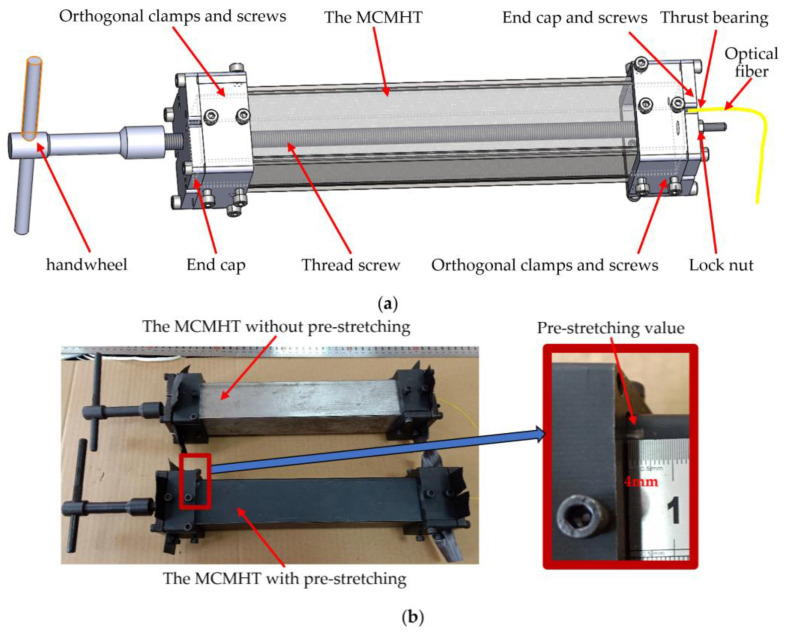
Structure of the MCMHT with the axial preload tool: (**a**) assembly schematic; (**b**) real fabricated MCMHTs.

The detailed fabrication processes of the MCMHT with the axial preload tool are as follows: (1) manufacturing the internal steel square tube and orthogonal steel plates, and the metal surfaces need to be roughened with wire wheels to increase the interfacial adhesive strength between the metal plate and the prepreg. Finally, metal surfaces are degreased and cleaned with acetone [32,33]. (2) Wrapping 5 unidirectional CFRP prepregs on the internal steel square tube by hand layup, and the fiber direction of the unidirectional prepreg is oriented in line with the axis of the internal steel square tube. (3) Placing the FBG strain sensor (FBGSS) in the middle of the face of the CFRP layers, and the FBG temperature sensor (FBGTS) placed nearby can be used for temperature compensation. The two optical fiber sensors are protected by a Teflon tube in the egress location. (4) Wrapping 5 other unidirectional CFRP prepregs, in the same manner as step 2, to make the total prepreg layers of 1 mm thickness. (5) Covering the wrapped internal steel square tube and sensors with the two orthogonal steel plates, which compose of an external steel square tube. (6) Clamping the external steel square tube with orthogonal clamps and screws at both ends to ensure enough contact between the steel-CFRP-steel sandwich walls for effective co-cure bonding. The MCMHT is assembled with [St/0_10_/St] symmetric tacking sequences. (7) Installing the axial preload tool to reduce the thermal residual strains/stresses by pre-compressing of the metal layers or pre-stretching of the CFRP layers. Because of the fastening of the CFRP layers at the end by the end caps and orthogonal clamps, and the design of the threaded screw motion and thrust bearing, the preload tool can apply compressive forces to the metal layers or tensile forces to the CFRP layers by rotating the handwheel. The assembly schematic of the MCMHT with the axial preload tool is shown in Figure 2. Two MCMHTs are fabricated in this paper, one of which is pre-stretched in the CFRP layers with 4 mm, as shown in Figure 2b.

**Figure 3:**
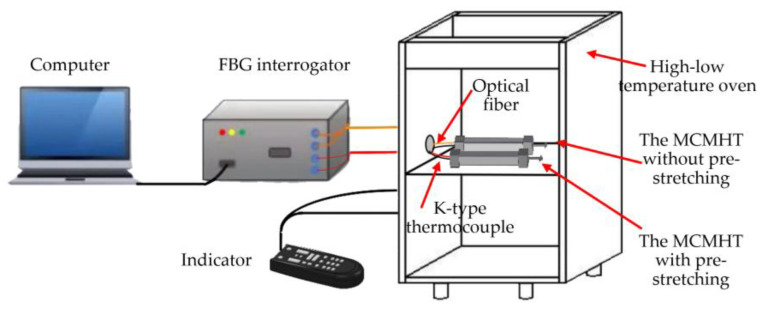
Co-cure experimental setup for the MCMHT with the axial preload tool.

The co-cure experimental setup for the MCMHT with the axial preload tool is shown in Figure 3. The two MCMHTs are put in the high-low temperature oven. A standard K-type thermocouple is fixed on the surface of each MCMHT with thermally conductive adhesive. Both signal wires of FBGs and thermocouples are fed through a specially reserved sealing hole in the oven wall and connected to the FBG interrogator and the thermocouple temperature indicator. The computer is used to record the Bragg wavelength shifts of FBGs by the cable connected to the FBG interrogator. The detailed experimental conditions are listed in Table 2.

For the fabrication of the co-cure bonding of MCMHTs with the axial preload tool, the manufacturer’s recommended cure cycle is used. The process is a typical curing cycle for thin CFRP/epoxy composite and is characterized by a heat-up ramp and dwell stages. The temperature is enhanced to the cure temperature (120 °C) in 1.5 h and held for 1.5 h. Finally, the MCMHTs are cooled to room temperature. During those stages, the adhesive bonding between the unidirectional CFRP prepregs and internal/external steel square tube is realized by the prepreg’s resin. During the cooling stage, thermal residual strains/stresses appear due to the different CTE between the steel and the composite.

### 2.3. Measurement of Strains through Optical Fiber Sensors

#### 2.3.1. Sensing Principle of FBG Sensor

The FBG is composed of a periodic distribution of the refractive index, which is made by ultraviolet exposure in the optical fiber core. When an incident broadband light passes through an FBG, a narrow-band light with a particular wavelength, called a Bragg wavelength, is reflected. The Bragg wavelength, $\lambda_{B}$, satisfies the Bragg scattering condition. It is expressed by the following equation [34]:

(1) $$\lambda_{B}=2\cdot n_{eff}\cdot \Lambda ,$$

The value of the Bragg wavelength depends on the effective refractive index of the fiber core, $n_{eff}$, and the grating period, Λ. However, when the FBG is subjected to axial strain ε or temperature changes ΔT, both the grating period and the effective refractive index change, and then result in the Bragg wavelength shift, $\Delta \lambda_{B}$. The Bragg wavelength variation which is sensitive to strain and temperature simultaneously can be expressed as:

(2) $$\Delta \lambda_{B}=\lambda_{B}\left(1-P_{e}\right)\varepsilon +\lambda_{B}\left(\alpha_{f}+\xi \right)\Delta T=K_{\varepsilon }\varepsilon +K_{T}\Delta T,$$

where $P_{e}$, $\alpha_{f}$, ξ are the effective photo-elastic coefficients, the thermal expansion coefficients, and the thermo-optic coefficients, respectively, and $K_{\varepsilon }$, $K_{T}$ are the strain sensitivity constants and the temperature sensitivity constants, respectively. The strain and temperature sensitivity constant of FBG sensors depend on the type of fibers. As $P_{e}$ has a typical value of 0.22 for fused silica [35], $K_{\varepsilon }$ is 1.2 pm/με in this paper without calibration for an FBG of a central wavelength of 1547 nm. However, $K_{T}$ requires a calibration procedure because FBG exhibits linear thermal-optic behavior only over a certain temperature range. The detailed calibration procedure can be seen in the next section.

**Figure 4:**
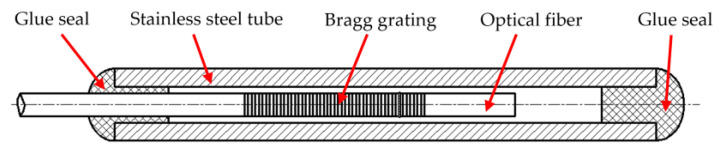
Encapsulated FBG for temperature measurement.

According to Equation (2), it can be found that the changes of FBG wavelength are proportional to axial strain and temperature. As consequence, a single FBG cannot avoid strain-temperature cross-sensitivity, as both strain and temperature induce a Bragg wavelength shift. Several techniques to achieve such discrimination are available in the literature [36]. In this paper, two separate FBG sensors are embedded into a structure to avoid FBG cross-sensitivity. The FBGTS is a 10 mm long FBG encapsulated in a stainless-steel tube as shown in Figure 4. Considering a normal optical fiber with an outer diameter of 0.125 mm, the inner and outer diameters of the stainless-steel tube are 0.2 mm and 0.4 mm: as small as possible to avoid affecting hybrid structural integrity. As consequence, the FBGTS only has relations with the temperature change theoretically. Therefore, Equation (2) can be simplified as [37]:

(3) $$\Delta \lambda_{B1}=K_{T1}\Delta T,$$

The FBGSS is a bare FBG with no treatment and is affected by axial strain and temperature. In this paper, the FBGTS is placed near to the FBGSS for the same temperature changes. Then the axial strain can be obtained from the measured wavelength shift by combining Equation (2) with Equation (3) [37].

(4) $$\varepsilon =\Delta \lambda_{B}/K_{\varepsilon }-\left(\Delta \lambda_{B1}K_{T}\right)/\left(K_{T1}K_{\varepsilon }\right),$$

#### 2.3.2. Temperature Calibration

Four FBGs (two FBGTSs and two FBGSSs) and a standard K-type thermocouple are fixed on an aluminum plate with a thermally conductive adhesive in an oven. It is ensured that the FBGs are in strain-free condition, so they respond to temperature change only. The Bragg wavelength shifts of FBGs are monitored by an FBG interrogator with a minimum resolution of 1 pm and a maximum sampling frequency of 4 kHz. The reliability of the encapsulated FBGTS must be confirmed before the calibration procedure. When the two FBGTSs are subjected to axial load at room temperature, no obvious wavelength shift is observed, so the two FBGTSs are considered as in strain-free condition. Both signal wires of FBGs and thermocouples are fed through the sealing strip of the oven door and connected to the FBG interrogator and the thermocouple temperature indicator. As the oven temperature is uniformly increased from 25 °C–200 °C, the results of the Bragg wavelength shifts are recorded by the computer. From the linear fitting results, the initial central wavelengths $K_{T}$ of the Four FBGs are listed in Table 3.

### 2.4. Experimental Setup of Modal Testing

**Figure 5:**
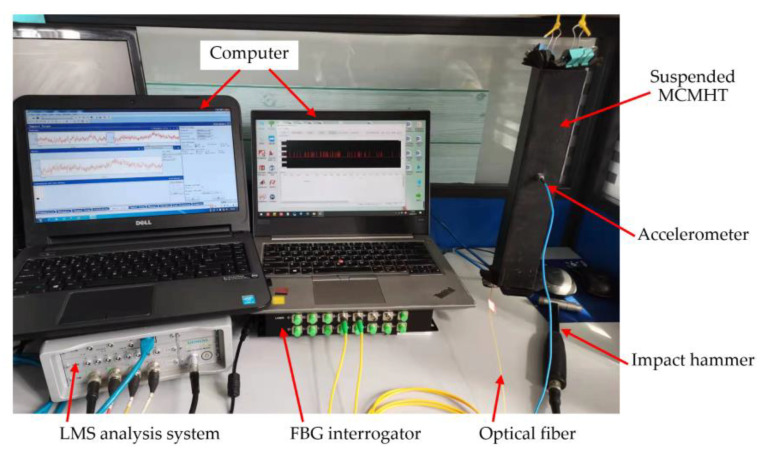
Experimental setup of modal testing.

Modal testing is performed to study the dynamic characteristics of the MCMHTs with different thermal residual strain states under vibrational excitation. According to the experimental equipment, as shown in Figure 5, the MCMHT with pre-stretching is suspended to emulate the free–free boundary. For comparison, the same experiment is done for the MCMHT without pre-stretching. A force transducer connected to the hammer is used to measure the force history of vibrations of the MCMHT caused by an impact hammer. With an accelerometer bonded on the surface, the acceleration response of the MCMHT is detected in a similar way. The excitation and response signals are subsequently acquired and analyzed by the LMS analysis system developed by Siemens company. Based on the obtained frequency response function (FRF), the modal parameters, including the first natural frequencies and damping ratio, can be processed by the modal analysis module. In order to obtain relatively accurate results, each MCMHT is measured with 7 excitation points along its length and each excitation point is applied 3 times to obtain the average FRF.

## 3. Evaluation of Thermal Residual Strains Based on Classical Lamination Theory

The analytical model considers thermal residual strains produced only during the cooling phase stage, based on the following assumptions [38]: (1) the CFRP layers are plane stresses; (2) each lamina has a unique and linearly elastic deformation; (3) perfect bonding occurs between layers without gaps, debonding and other defects. Classical lamination theory is applied to predict the laminate properties of orthotropic continuous fiber laminated composites.

### 3.1. Material Properties of the Laminate with Arbitrary Lamina Orientation Angle

The stiffness and transformation matrices for predicting the engineering constants of the CFRP layers are expressed as follows [39]. The stiffness matrix [Q] and transformation matrix [T] are:

(5) $$\left[Q\right]=\left[\begin{array}{ccc} \frac{E_{1}}{1-\nu_{12}v_{21}} & \frac{v_{12}E_{2}}{1-\nu_{12}v_{21}} & 0\\ \frac{v_{21}E_{1}}{1-\nu_{12}v_{21}} & \frac{E_{2}}{1-v_{12}v_{21}} & 0\\ 0 & 0 & G_{12} \end{array}\right],$$

(6) $$\left[T\right]=\left[\begin{array}{ccc} \cos^{2}\theta & \sin^{2}\theta & 2\sin\theta \cos\theta \\ \sin^{2}\theta & \cos^{2}\theta & -2\sin\theta \cos\theta \\ -\sin\theta \cos\theta & \sin\theta \cos\theta & \cos^{2}\theta -\sin^{2}\theta \end{array}\right],$$

The stiffness for angled lamina is:

(7) $${\left[\overset{¯}{Q}]=[T\right]}^{-1}\left[Q\right]\left[\begin{array}{ccc} 1 & 0 & 0\\ 0 & 1 & 0\\ 0 & 0 & 2 \end{array}\right]\left[T\right],$$

where $E_{1}$, $E_{2}$, $G_{12}$, $\nu_{12}$, $v_{21}$, θ represent longitudinal Young’s modulus, transverse Young’s modulus, shear modulus, major Poisson’s ratio, minor Poisson’s ratio, and lamina orientation angle, respectively.

The extensional stiffnesses matrix [A], strain-curvature coupling stiffness matrix [B], and bending stiffness matrix [D] for laminate are given by:

(8) $$\left[A\right]=\sum_{k=1}^{N}{\left({\overset{¯}{Q}}_{ij}\right)}_{k}\left(z_{k}-z_{k-1}\right)=\sum_{k=1}^{N}{\left({\overset{¯}{Q}}_{ij}\right)}_{k}t_{k},,$$

(9) $$[B]=\frac{1}{2}\sum_{k=1}^{N}({\bar{C}}_{ij}{)}_{k}(z_{k}^{2}-z_{k-1}^{2})=\sum_{k=1}^{N}({\bar{C}}_{ij}{)}_{k}t_{k}{\bar{z}}_{k},$$

(10) $$[D]=\frac{1}{3}\sum_{k=1}^{N}({\bar{C}}_{ij}{)}_{k}(z_{k}^{3}-z_{k-1}^{3})=\sum_{k=1}^{N}({\bar{C}}_{ij}{)}_{k}(t_{k}z_{k}^{-2}+\frac{t_{k}^{3}}{12}),$$

where $z_{k}$ and $t_{k}$ represent the vertical position of the kth lamina from the mid-plane and thickness of the kth lamina, respectively. N represents the total number of layers of the laminate. The subscript i,j=1,2,…,6, whose meaning can be found in any textbook of composite mechanics.

For symmetric laminate, the effective longitudinal Young’s modulus of the laminate $E_{x}$, the effective transverse Young’s modulus of the laminate $E_{y}$, the effective laminate in-plane shear modulus $G_{xy}$, and the effective laminate longitudinal Poisson’s ratio $\nu_{xy}$, are defined as:

(11) $$E_{x}=\frac{\sigma_{x}}{\varepsilon_{x}^{0}}=\frac{A_{11}A_{22}-A_{12}{\;}^{2}}{t_{\;}A_{22}},$$

(12) $$E_{y}=\frac{\sigma_{y}}{\varepsilon_{y}^{0}}=\frac{A_{11}A_{22}-A_{12}{\;}^{2}}{t_{\;}A_{11}},$$

(13) $$G_{xy}=\frac{\tau_{xy}}{\Upsilon_{xy}^{0}}=\frac{A_{66}}{t_{\;}},$$

(14) $$\nu_{xy}=\frac{A_{12}}{A_{22}},$$

(15) $$\nu_{yx}=\frac{A_{12}}{A_{11}},$$

where t represents the thickness of the laminate.

### 3.2. Strains in Metal-CFRP-Metal Hybrid Structure

**Figure 6:**
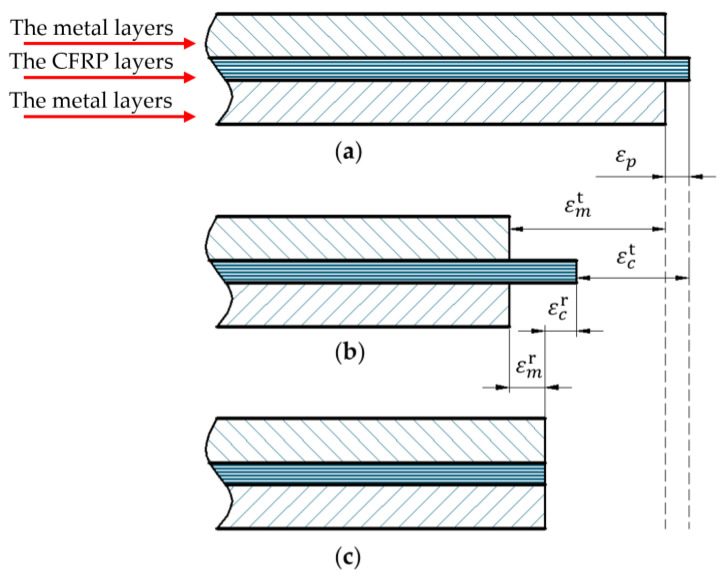
Schematic of thermal residual strains: (**a**) no strain state; (**b**) ideal state; (**c**) final state.

Figure 6 is the evaluation of thermal residual strains in metal-CFRP-metal hybrid structure after the co-cure bonding process. Figure 6a represents that no strain appears in the dwell stage except for the pre-stretching strain $\varepsilon_{p}$. Figure 6b shows an ideal state where there is no interface interaction between the metal and CFRP layers. $\varepsilon_{m}^{t}$ and $\varepsilon_{c}^{t}$ represent the thermal strain in the metal layer and the CFRP layer, respectively. As shown in Figure 6c, $\varepsilon_{m}^{r}$ and $\varepsilon_{c}^{r}$ represent the thermal residual strain in the metal layer and the CFRP layer, respectively.

Based on the unique deformation, total strains of the two materials, which are composed of pre-stretching strain, thermal residual strain, and thermal strain, can be described as:

(16) $$\varepsilon_{m}^{r}+\varepsilon_{m\;}^{t}=\varepsilon_{p}+\varepsilon_{c}^{r\;}+\varepsilon_{c}^{t},$$

Taking into account the isotropic metal layer and the anisotropic CFRP layer, the above equation can be expressed as:

(17) $$[\varepsilon_{m}]+\left[\alpha_{m}\right]\Delta T\;=\left[\varepsilon_{p}\right]+\left[\varepsilon_{c}\right]+[\alpha_{c}]\Delta T,$$

where $\left[\varepsilon_{m}\right]=\left[\begin{array}{c} \varepsilon_{m}\\ \varepsilon_{m} \end{array}\right]$, $\left[\varepsilon_{p}\right]=\left[\begin{array}{c} \varepsilon_{p1}\\ \varepsilon_{p2} \end{array}\right]$, $\left[\varepsilon_{c}\right]=\left[\begin{array}{c} \varepsilon_{c1}\\ \varepsilon_{c2} \end{array}\right]$, $\left[\alpha_{m}\right]=\left[\begin{array}{c} \alpha_{m}\\ \alpha_{m} \end{array}\right]$ and $\left[\alpha_{c}\right]=\left[\begin{array}{c} \alpha_{1}\cos^{2}\theta +\alpha_{2}\sin^{2}\theta \\ \alpha_{1}\sin^{2}\theta +\alpha_{2}\cos^{2}\theta \end{array}\right]$.

In which $\varepsilon_{m}$, $\varepsilon_{c1}$, $\varepsilon_{c2}$ represent the strains of the steel, and CFRP layer in the axial and transverse directions, respectively. $\varepsilon_{p1}$, $\varepsilon_{p2}$ represent the pre-stretching strains of the CFRP layer in the axial and transverse directions, respectively. $\alpha_{m}$, $\alpha_{c1}$, $\alpha_{c2}$ denote the CTEs of steel, and CFRP layers in the axial and transverse direction, respectively. ΔT denotes the temperature difference at different phases.

According to the force equilibrium equation from the mechanics of materials, residual strains in the MCMHT can be described as:

(18) $$A_{m}E_{m}[\varepsilon_{m}]+A_{c}\left[C\right][\varepsilon_{c}]=0,$$

where $[C]=\left[\begin{array}{cc} \frac{E_{x}}{1-\nu_{\mathrm{xy}}v_{\mathrm{yx}}} & \frac{\nu_{\mathrm{xy}}E_{y}}{1-\nu_{\mathrm{xy}}v_{\mathrm{yx}}}\\ \frac{v_{\mathrm{yx}}E_{x}}{1-\nu_{\mathrm{xy}}v_{\mathrm{yx}}} & \frac{E_{y}}{1-\nu_{\mathrm{xy}}v_{\mathrm{yx}}} \end{array}\right]$.

In which $A_{m}$ and $A_{c}$ represent the cross-sectional areas of steel and CFRP layers, respectively. $E_{m}$ and [C] represent Young’s modulus of steel and the stiffness matrix of CFRP layers, respectively.

Then, from Equations (17) and (18), the thermal residual strain in the CFRP layers can be expressed by:

(19) $$\left[\varepsilon_{c}\right]={\left[A\right]}^{-1}\left(\left[B\right]\;\Delta T-\left[\varepsilon_{p}\right]\right),$$

where $[A]=\left[\begin{array}{cc} 1+\frac{E_{x}A_{c}}{E_{m}A_{m}\left(1-\nu_{\mathrm{xy}}v_{\mathrm{yx}}\right)} & \frac{\nu_{\mathrm{xy}}E_{y}A_{c}}{E_{m}A_{m}\left(1-\nu_{\mathrm{xy}}v_{\mathrm{yx}}\right)}\\ \frac{v_{\mathrm{yx}}E_{x}A_{c}}{E_{m}A_{m}\left(1-\nu_{\mathrm{xy}}v_{\mathrm{yx}}\right)} & 1+\frac{E_{y}A_{c}}{E_{m}A_{m}\left(1-\nu_{\mathrm{xy}}v_{\mathrm{yx}}\right)} \end{array}\right]$ and $\left[B\right]=\left[\alpha_{s}\right]-\left[\alpha_{c}\right]$.

## 4. Measurement Results and Discussion

### 4.1. Comparison of Theoretical Calculation with Measurement by the FBGSS

**Figure 7:**
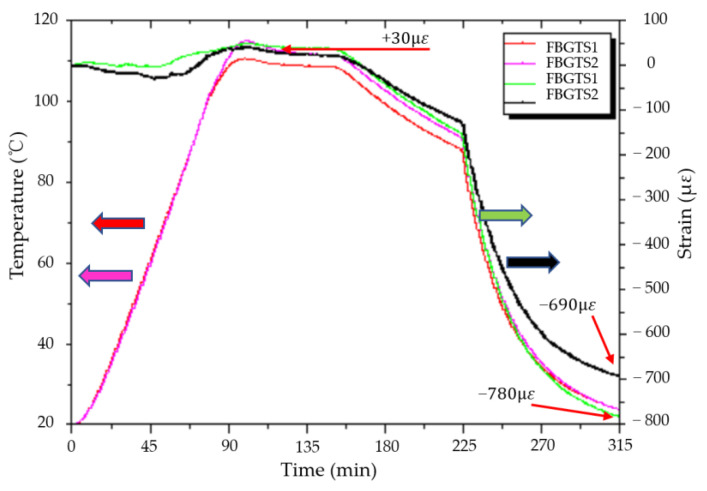
Temperature and strain history for the MCMHTs with and without pre-stretching during the co-cure bonding process.

The plot of temperature and strain history for the MCMHTs with and without pre-stretching during the co-cure bonding process is given in Figure 7. Simultaneous measurements of temperatures and strains during the co-cure bonding process are performed by the FBG sensors in real-time. It can be observed that the tendency of the temperature and strain measured for the MCMHTs with and without pre-stretching is consistent. The temperature measured by the FBGTS has good agreement with the cure cycle applied by the high-low temperature oven.

The trend of strain history is also the same for the two MCMHTs, but their values are slightly different due to the pre-stretching strain. The residual strains of the CFRP layers transform from tensile strains to compressive strains during the dwell and cooling stages. As one of the MCMHTs is pre-stretched in the CFRP layers as shown in Figure 2b, the pre-stretching strain in this case is 100 με, taking into account the slippage between the CFRP layers and the axial preload tool. Table 4 reports the comparison of the residual strains for the two MCMHTs obtained by the FBGSS and theoretical calculation. Considering that the thermal tensile strain is 30 με, it is observed that the residual compression strains for the MCMHT reduce from 810 με to 720 με, obtained by the FBGSS through pre-stretching of the CFRP layers. Indeed, it can also be reduced by pre-compressing of the metal layer because of the interaction between the CFRP and the metal. The important point is that the axial preload tool proposed in this paper can be used either clockwise or counterclockwise to achieve the pre-stretching of the CFRP layers or the pre-compressing of the metal layers. The measured results and theoretical calculations verify that the residual strains of the MCMHT can be modified by the axial preload tool. Furthermore, the residual strain can theoretically be eliminated by suitable preloading.

### 4.2. The Dynamic Characteristics of the MCMHT

**Figure 8:**
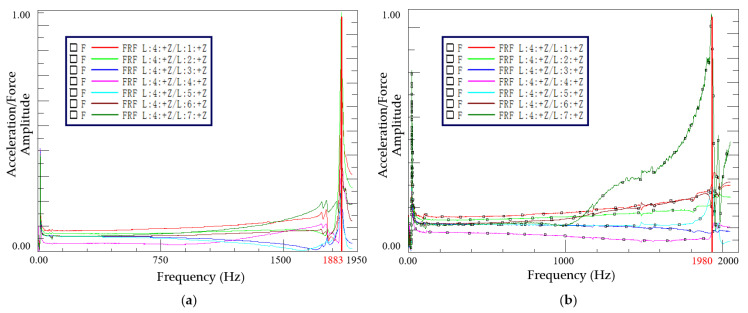
Acceleration FRFs of 7 excitation points for the MCMHTs: (**a**) without pre-stretching; (**b**) with pre-stretching.

Acceleration FRFs of 7 excitation points for the MCMHTs with different thermal residual strain states are processed by the LMS analysis system as shown in Figure 8. Dynamic characteristics of the MCMHTs from the modal analysis module are given in Table 5. It is observed that the first natural frequency of the MCMHT with pre-stretching is 5.2% higher than that without pre-stretching. Nevertheless, the damping of the MCMHT with pre-stretching decrease by 11.5% compared to the MCMHT without pre-stretching. The modal testing results imply that the reduction of residual strain increases the natural frequency of the MCMHT, but is detrimental to the damping capability of the MCMHT.

## 5. Conclusions

The axial preload tool is proposed to reduce the thermal residual strains/stresses during the co-cure bonding process by pre-compressing of the metal layers or pre-stretching of the CFRP layers. Residual strain determination by embedded optical fiber sensors in metal-composite hybrid structures is presented. Thermal residual strain results obtained from FBG sensors are compared with those obtained from theoretical calculations. Moreover, the dynamic characteristics of the MCMHTs with different stress states are compared. For future work, the embedded FBG sensors can be used for real-time structural health monitoring of composite structures, based on the multiplexing capability of FBG sensing technology. For example, damage such as microcracks, delamination at interfaces, or crushing of polymer matrix can reduce stiffness overall. By the embedded FBG sensors array, it is possible to determine the initiation, size, and location of the damage. The following conclusions can be drawn from the results obtained:

1. To reduce thermal residual strain, the proposed axial preload tool can apply compressive forces to the metal layers or tensile forces to the CFRP layers by rotating the handwheel. This shows the axial preload tool can change the strain state of the metal-composite hybrid structure;
2. Thermal residual strain of the metal-composite hybrid structure obtained from embedded optical fiber sensors show good agreement with the theoretical calculation based on classic laminate theory;
3. The modal testing results imply that the reduction of residual strain increases the natural frequency of the metal-composite hybrid structure, but is detrimental to its damping capability. This shows that the intrinsic properties of the metal-composite hybrid structure can be modified by the proposed axial preload tool.

## Figures and Tables

**Table 1:** Material properties of the unidirectional CFRP prepreg and the steel square tube.

Material Properties	USN 10000/T300 Prepreg	AISI 1045
Longitudinal modulus, E_1_ (GPa)	137	200
Transverse modulus, E_2_ (GPa)	9	200
Shear modulus, G_12_ (GPa)	3.78	80
Major Poisson’s ratio, ν_12_	0.28	0.29
Longitudinal CTE, α_1_ (10^−6^/°C)	−0.5	11
Transverse CTE, α_2_ (10^−6^/°C)	27	11
Density (g/cm^3^)	1.76	7.85

**Table 2:** The detailed experimental conditions.

Experimental Condition	MCMHT without Pre-Stretching	MCMHT with Pre-Stretching
Material	Unidirectional CFRP prepreg and AISI 1045	
Curing equipment	The high-low temperature oven	
Sensor	FBGTS1 and FBGSS1	FBGTS2 and FBGSS2
Axial preload tool	Without pre-stretching	With pre-stretching of 4 mm

**Table 3:** The temperature sensitivity constants of the four FBGs.

FBG	Initial Central Wavelength/nm	$K_{T}/\mathrm{pm}/{}^{\circ}C$	Fitting Linear Correlation Coefficient
FBGTS1	1546.914	11.80	99.95%
FBGTS2	1546.860	11.84	99.91%
FBGSS1	1537.041	11.20	99.84%
FBGSS2	1546.936	11.07	99.82%

**Table 4:** Comparison of the residual compression strains for the two MCMHTs obtained by the FBGSS and theoretical calculation.

MCMHT	by the FBGSS/με	by Theoretical Calculation/με	Relative Difference
With pre-stretching	720	659.2	−8.4%
Without pre-stretching	810	759.2	−6.2%

**Table 5:** Dynamic characteristics of the MCMHTs from the modal analysis module.

Characteristics	without Pre-Stretching	with Pre-Stretching	Relative Difference
Natural frequency/Hz	1883	1980	5.2%
Damping ratio	0.96%	0.85%	−11.5%

## Data Availability

Not applicable.
